# Fisetin alleviates zinc overload-induced intestinal injury by inhibiting ferroptosis and remodeling gut microbiota in weaned piglets

**DOI:** 10.1186/s40104-026-01481-0

**Published:** 2026-08-09

**Authors:** Feifei Huang, Yuhang Deng, Mohan Zhou, Huangen Xu, Jie Feng

**Affiliations:** 1 Key Laboratory of Nutrition and Breeding for High-Quality Animal Products of Zhejiang Province, College of Animal Science, Zhejiang University, Hangzhou, 310058 China; 2 Key Laboratory of Animal Molecular Nutrition Ministry of Education, College of Animal Science, Zhejiang University, Hangzhou, 310058 China; 3 Research Center of Zhejiang Kesheng Feed Co., Ltd., Shaoxing, 312000 Zhejiang China

**Keywords:** Ferroptosis, Fisetin, Intestinal health, Weaned piglets, Zinc overload

## Abstract

**Background:**

High doses of zinc oxide (ZnO) effectively prevent post-weaning diarrhea and promote growth in weaned piglets, but raise concerns over intestinal injury, metabolic disorders, and risks of fostering bacterial resistance. This study aimed to identify non-chelating polyphenols capable of mitigating zinc toxicity and to elucidate their protective mechanisms, with emphasis on ferroptosis inhibition.

**Results:**

Network toxicology predicted ferroptosis as a central mechanism in zinc-induced intestinal injury, which was confirmed in intestinal epithelial cells where zinc overload specifically induced ferroptosis without activating apoptosis, necroptosis, or autophagy. From multiple polyphenols, fisetin (FIS) was identified as a non-chelating candidate that alleviated zinc-induced cytotoxicity, preserved tight junction proteins, and activated the Nrf2-GPX4 axis to inhibit ferroptosis in vitro. In zinc-overloaded weaned piglets, FIS supplementation maintained the growth-promoting effects of high-dose zinc while ameliorating intestinal damage. FIS also attenuated oxidative stress, alleviated inflammation, and inhibited ferroptosis in the jejunum. Furthermore, FIS remodeled the gut microbiota, enriching beneficial taxa *Romboutsia* (positively correlated with growth performance) and *Clostridium_sensu_stricto_1* (positively correlated with the p-Nrf2/Nrf2 ratio), while suppressing the zinc-enriched *Anaerovibrio* (negatively correlated with GPX4 protein expression). FIS also shifted microbial metabolic pathways toward amino acid and terpenoid metabolism, potentially contributing to the observed ferroptosis defense.

**Conclusion:**

FIS alleviates high-dose ZnO-induced intestinal injury in weaned piglets through dual modulation, activating the Nrf2-GPX4 axis to inhibit ferroptosis and remodeling the gut microbiota to reinforce this defense. By preserving the growth benefits of zinc while mitigating its toxicity, FIS represents a promising nutritional strategy for sustainable swine production.

**Supplementary Information:**

The online version contains supplementary material available at 10.1186/s40104-026-01481-0.

## Introduction

The weaning period in piglets involves multiple stressors, resulting in diarrhea and impaired growth [1]. High-dose zinc oxide (ZnO, 2,000–3,000 mg/kg) effectively controls post-weaning diarrhea and promotes growth [2], but its use raises concerns due to its double-edged nature. Beyond benefits, long-term (e.g., > 2 weeks) or high-dose ZnO disrupts mineral homeostasis (e.g., iron, copper), and induces hepatic mitochondrial dysfunction [3, 4].

In addition to the liver, the intestine is the primary site for zinc absorption and a critical interface for immune and microbial homeostasis [5, 6]. Accumulating evidence indicates that zinc overload markedly alters the gut microbiota composition in weaned piglets [7, 8]. Gut dysbiosis is known to increase intestinal permeability, promote bacterial translocation, and trigger systemic inflammation [9, 10]. Consequently, intestinal dysregulation is a pivotal driver of systemic disturbances.

At the cellular level, emerging evidence from lung and vascular endothelial cells suggests that zinc overload induces ferroptosis [11, 12], a form of cell death driven by iron‑dependent lipid peroxidation. It is plausible that zinc overload similarly triggers ferroptosis in the intestine. Such ferroptosis could disrupt epithelial integrity and exacerbate intestinal damage through interaction with gut microbiota dysbiosis. Notably, certain polyphenols have been reported to inhibit ferroptosis and modulate gut microbiota [13, 14]. Polyphenols possess antioxidant, anti-inflammatory, and barrier-protective properties [15]. However, some polyphenols chelate metal ions [16], potentially interfering with zinc homeostasis. Thus, identifying non-chelating polyphenols that can counteract ferroptosis and remodel the microbiota without disturbing zinc homeostasis represents a novel and strategic approach.

Accordingly, this study aimed to identify a non-chelating polyphenol from a library of 22 candidates and to evaluate its protective effects against zinc overload-induced intestinal damage in vitro and in vivo. Specifically, we tested whether the selected polyphenol could inhibit ferroptosis, restore intestinal barrier function, and ameliorate microbiota dysbiosis.

## Materials and methods

### Acquisition of zinc- and intestinal injury-related target genes

Potential zinc targets were queried from the GeneCards (Weizmann Institute of Science, Rehovot, Israel) and CTD (North Carolina State University, Raleigh, NC, USA) databases using "zinc" as a keyword. Similarly, intestinal injury-related genes were retrieved from the OMIM (Johns Hopkins University, Baltimore, MD, USA) and the GeneCards databases by searching “intestinal injury”. Duplicates were removed, and the targets from GeneCards and CTD were refined by applying a top 10% relevance score and interaction count ≥ 10, respectively. These thresholds were chosen because they are commonly used in network toxicology to balance sensitivity and specificity, retaining highly relevant targets without excessive noise [17, 18]. The final step involved identifying the intersection between the zinc and intestinal injury target sets using online tool Bioinformatics (Bioinformatics & Evolutionary Genomics group, Ghent University, Belgium).

### Construction and analysis of the protein–protein interaction (PPI) network for hub gene identification

To investigate the mechanisms of zinc-induced intestinal injury, the overlapping targets between zinc and intestinal injury were used to construct a PPI network using the STRING database (version 11.5; Swiss Institute of Bioinformatics, Lausanne, Switzerland), with the organism set to "*Sus scrofa*" and a medium confidence interaction score of 0.400. The resulting network was then imported into Cytoscape (v3.10.1; Cytoscape Consortium, San Diego, CA, USA) for topological analysis. The cytohubba, MCODE and CytoNAC plugins in Cytoscape were applied to identify the core genes.

### Functional analysis of potential targets

To explore the biological functions and pathways involved in zinc-induced intestinal injury, Gene Ontology (GO) and Kyoto Encyclopedia of Genes and Genomes (KEGG) enrichment analyses were conducted using the DAVID database (version 6.8; National Institute of Allergy and Infectious Diseases, Bethesda, MD, USA), with organism set to *Sus scrofa*. GO annotation covered three categories: biological process (BP), cellular component (CC), and molecular function (MF). Enrichment results were visualized using the Bioinformatics online platform.

### Polyphenol selection

A library of 22 polyphenols (see Table S1 for the complete list and references; Fig. S1 for chemical structures) was selected for initial screening. The selection was based on their antioxidant and anti-inflammatory properties, reported efficacy against metal-induced toxicity, structural diversity and commercial availability. All 22 polyphenols in cell experiments were purchased from MedchemExpress (Monmouth Junction, NJ, USA).

### Cell culture and treatment

IPEC-J2 cells were grown in DMEM (#C11965500BT, Gibco, Grand Island, NY, USA) with 10% (v/v) fetal bovine serum (#10099141C, Gibco, Australia) and 1% penicillin–streptomycin (#15140122, Gibco, Grand Island, NY, USA) at 37 °C under 5% CO_2_. The experimental unit was an individual well of a culture plate. Each treatment was applied to separate wells, and each well was considered an independent replicate. For the screening and mechanism studies, cells were co-treated with 200 μmol/L ZnSO_4_ (#Z0251, Sigma-Aldrich, St. Louis, MO, USA) and individual polyphenols (MedchemExpress, Monmouth Junction, NJ, USA) at various concentrations (5, 10, 20, and 30 μmol/L), or specific inhibitors, including ferrostatin-1 (Fer-1, ferroptosis inhibitor, #HY-100579; MedchemExpress, Monmouth Junction, NJ, USA) and chloroquine (CQ, autophagy inhibitor, #HY-17589A; MedchemExpress, Monmouth Junction, NJ, USA) for 2 h.

### Cell viability

Cell viability was assessed using the Cell Counting Kit-8 (CCK-8) assay (#HY-K0301, MedchemExpress, Monmouth Junction, NJ, USA). After treatment, 10 μL CCK-8 reagent was added per well and incubated for 2 h. Absorbance at 450 nm was recorded with a microplate reader (Bio-Rad, Hercules, CA, USA), and viability was normalized to the control.

### Intracellular Zn^2+^ level

Treated cells in 6-well plates were harvested, washed with phosphate-buffered saline (PBS), and loaded with 1 μmol/L FluoZin™−3, AM (#F24195, Invitrogen, Carlsbad, CA, USA) at 37 °C for 1 h. Following a final wash, cells were resuspended in PBS, and the fluorescence was measured (Ex/Em = 494/516 nm) with a microplate reader.

### Immunofluorescence staining

The localization and expression of tight junction proteins were assessed by immunofluorescence. Following treatments, cells were fixed with 4% paraformaldehyde for 20 min, permeabilized with 0.1% Triton X-100 (#GC204003, Servicebio, Wuhan, China) for 10 min, and blocked with 5% BSA (#GC305010, Servicebio, Wuhan, China) for 30 min at room temperature. Primary antibody incubation (ZO-1, #21773-1-AP; Occludin, #66378-1-Ig; Claudin-1, #13050-1-AP; Proteintech, Wuhan, China) was performed overnight at 4 °C. Cells were then incubated with Cy3 or FITC-conjugated secondary antibodies (#GB22303, #GB22301, #GB21303; Servicebio, Wuhan, China) for 1 h at room temperature in the dark. Nuclei were stained with DAPI (#62248; Thermo Fisher Scientific, Waltham, MA, USA), and images were acquired with a SpinSR super-resolution confocal microscope (Olympus, Tokyo, Japan).

### Animal experiment

All animal procedures were approved by the Institutional Animal Care and Use Committee of Zhejiang University (AP code: ZJU20240807). The experiment was conducted at Zhejiang Kesheng Ecological Agriculture Co., Ltd. (Shaoxing, China). Thirty weaned piglets (25-day-old, Duroc × Landrace × Yorkshire) were housed individually in stainless steel pens (1.80 m × 1.10 m) at 25–28 °C with free access to feed and water, and their health status was monitored twice daily. The experimental unit was an individual piglet. Piglets were randomly divided into three groups: CON group (basal diet, 100 mg/kg Zn), Zn overload group (basal diet +1,500 mg/kg Zn as ZnO), Zn overload + fisetin (FIS) group (Zn overload diet supplemented with 200 mg FIS per kg of diet). FIS was purchased from YuanYe (Shanghai, China). Dietary supplementation was used to minimize handling stress, and the FIS dosage was based on reported levels of structurally similar flavonoids (e.g., quercetin, proanthocyanidins, curcumin) in swine studies [19–21]. The basal diet was formulated to meet NRC (2012) [22] nutrient requirements (detailed composition in Table S2). Initial body weight and final body weight (FBW) as well as feed intake were recorded to calculate average daily gain (ADG) and feed-to-gain ratio (F/G). On d 14, six piglets from each group were euthanized, and jejunum and cecal content samples were collected for analysis.

### Zn^2+^ measurement in serum and jejunum

Zn^2+^ concentrations in serum and jejunal homogenates were measured using a commercial assay kit (#E011-1-1, Nanjing Jiancheng Bioengineering Institute, Nanjing, China).

### Hematoxylin and eosin (H&E) staining

Jejunal morphological damage was evaluated using H&E staining. Briefly, tissue samples were fixed in 4% paraformaldehyde, dehydrated through a graded ethanol series, cleared in xylene, and embedded in paraffin. Sections of 5 μm thickness were cut, deparaffinized, rehydrated, and stained with hematoxylin and eosin. The morphological structure of the jejunum was examined and imaged under a light microscope (Eclipse E100, Nikon, Tokyo, Japan).

### Transmission electron microscopy (TEM)

For ultrastructural observation, fresh jejunal tissues were fixed in 2.5% glutaraldehyde, post-fixed in 1% osmium tetroxide, and dehydrated through a graded ethanol series (50%, 70%, 80%, 90%, 95% and 100%) and pure acetone. The samples were embedded in Epon 812 resin (Structure Probe, Inc., West Chester, PA, USA), and ultrathin sections were prepared. Following staining with uranyl acetate and lead citrate, the sections were observed under a transmission electron microscope (HT-7820, Hitachi, Tokyo, Japan).

### Oxidative stress level

The intracellular accumulation of reactive oxygen species (ROS) was detected by using the CM-H2DCFDA (Invitrogen, Carlsbad, CA, USA) according to previous study [23]. For piglet jejunum, frozen sections were prepared and incubated with DCFH-DA (#HY-D0940, MedchemExpress, Monmouth Junction, NJ, USA) at 37 °C for 30 min in the dark. After washing, ROS fluorescence was visualized and captured using a fluorescence microscope (Eclipse Ci, Nikon, Tokyo, Japan), with images analyzed by ImageJ software (National Institutes of Health, Bethesda, MD, USA).

Malondialdehyde (MDA; #A003-1-2, #A003-4-1), total antioxidant capacity (T-AOC; #A015-2-1), and glutathione (GSH; #A006-2-1) were quantified with commercial kits (Nanjing Jiancheng Bioengineering Institute, Nanjing, China). Measurements were performed in cell lysates, and piglet jejunal homogenates according to the manufacturer's protocols.

Lipid peroxidation level in IPEC J2 cells was evaluated using the BODIPY™ 581/591 C11 kit (#S0043S; Beyotime Biotechnology, Shanghai, China). Cells were stained with 4 μmol/L of the BODIPY C11 probe in PBS at 37 °C for 20 min, washed twice, resuspended in 500 μL PBS, and analyzed on a flow cytometer (FACS Calibur, BD Biosciences, San Jose, CA, USA) using the FITC channel, with at least 10,000 cells per sample.

### Intestinal permeability markers

Serum diamine oxidase (DAO; #A088-3-1) activity and D‑lactate (#A019-3-2) levels were measured using commercial kits (Nanjing Jiancheng Bioengineering Institute, Nanjing, China) according to the manufacturer’s protocols.

### Reverse transcription quantitative polymerase chain reaction (RT-qPCR)

Total RNA from both cell cultures and jejunal tissues was extracted from cells using the SteadyPure RNA extraction kit (#AG21024, Accurate Biology, Changsha, China), and cDNA was synthesized using Evo M − MLV RT Mix kit (#AG11728, Accurate Biology, Changsha, China). Quantitative PCR was performed using SYBR Green Pro Taq HS qPCR kit (#AG11701, Accurate Biology, Changsha, China) on a CFX384 Real-Time PCR System (Bio-Rad, Hercules, CA, USA). The mRNA expression levels of target genes were normalized to the level of β-actin, and relative quantification was calculated using the 2^–ΔΔCt^ method. All primer sequences used are provided in Table S3.

### Inflammatory cytokines

The mRNA expression levels of tumor necrosis factor-alpha (*TNF-α*), interleukin (*IL*)-8, and *IL-10* in cells were determined by RT-qPCR.

In animal experiments, the protein concentrations of TNF-α (#H052-1-1), IL-1β (#H002-1-1), and IL-6 (#H007-1-1) of piglet jejunum were measured using commercial ELISA kits (Nanjing Jiancheng Bioengineering Institute, Nanjing, China).

### Western blotting

Western blotting was carried out as described [23]. Total or nuclear proteins were extracted from cells or jejunal tissues, resolved by sodium dodecyl sulfate–polyacrylamide gel electrophoresis (SDS-PAGE), transferred to polyvinylidene fluoride (PVDF) membranes and blocked. After blocking, membranes were incubated overnight at 4 °C with primary antibodies against β-actin (#M1210-2; HUABIO, Hangzhou, China), sequestosome‑1 (p62, #HA721171; HUABIO, Hangzhou, China), microtubule‑associated protein 1 light chain 3B (LC3B, #db15555; Diagbio, Hangzhou, China), cleaved caspase 3 (#ET1602-47; HUABIO, Hangzhou, China), glutathione peroxidase 4 (GPX4, #T56959; Abmart, Shanghai, China), nuclear factor erythroid 2-related factor 2 (Nrf2, #16396-1-AP; Proteintech, Wuhan, China), phosphorylated Nrf2 (p-Nrf2, #ab76026; Abcam, Cambridge, MA, USA), heme oxygenase-1 (HO-1, #HA721854; HUABIO, Hangzhou, China), zonula occludens-1 (ZO-1, #21773-1-AP; Proteintech, Wuhan, China) Claudin-1 (#13050-1-AP; Proteintech, Wuhan, China), and Histone H3 (#GB12102; Servicebio, Wuhan, China). After incubation with horseradish peroxidase (HRP)-conjugated secondary antibodies, chemiluminescent signals were detected. Protein expressions were quantified using Image Lab software (Bio-Rad, Hercules, CA, USA), with phosphorylated proteins normalized to their total counterparts, nuclear proteins normalized to histone H3, and total proteins normalized to β-actin.

### 16S ribosomal DNA (rDNA) sequencing and bioinformatics analysis

Microbial DNA was extracted from cecal contents, and the V3–V4 region of 16S rRNA gene was amplified (primers 338F/806R) and sequenced on an Illumina NovaSeq 6000 (Novogene, Beijing, China). Raw data were processed in QIIME2 (v2023.2) with DADA2 for quality control and ASV generation. Taxonomy was assigned using SILVA (release 138). Linear discriminant analysis effect size (LEfSe) identified differential taxa, and Phylogenetic Investigation of Communities by Reconstruction of Unobserved States (PICRUSt) predicted functional profiles with enriched GO and KEGG pathways. Spearman correlations between genus abundance and host phenotypes were analyzed.

### Statistical analysis

Statistical analysis was performed using SPSS software (version 22.0, IBM Corp., Armonk, NY, USA). Data were presented as mean ± standard deviation (SD). For comparisons between two groups, an independent-sample *t*-test was employed. For multiple group comparisons, one-way analysis of variance (ANOVA) was performed. When variances were homogeneous (*P* > 0.05), Duncan’s multiple range test was used for post-hoc comparisons; when variances were not homogeneous (*P* < 0.05), Dunnett’s T3 test was applied. A value of *P* < 0.05 was considered statistically significant.

## Results

### Network toxicology implicates multiple cell death pathways in zinc-induced intestinal injury

**Fig. 1:**
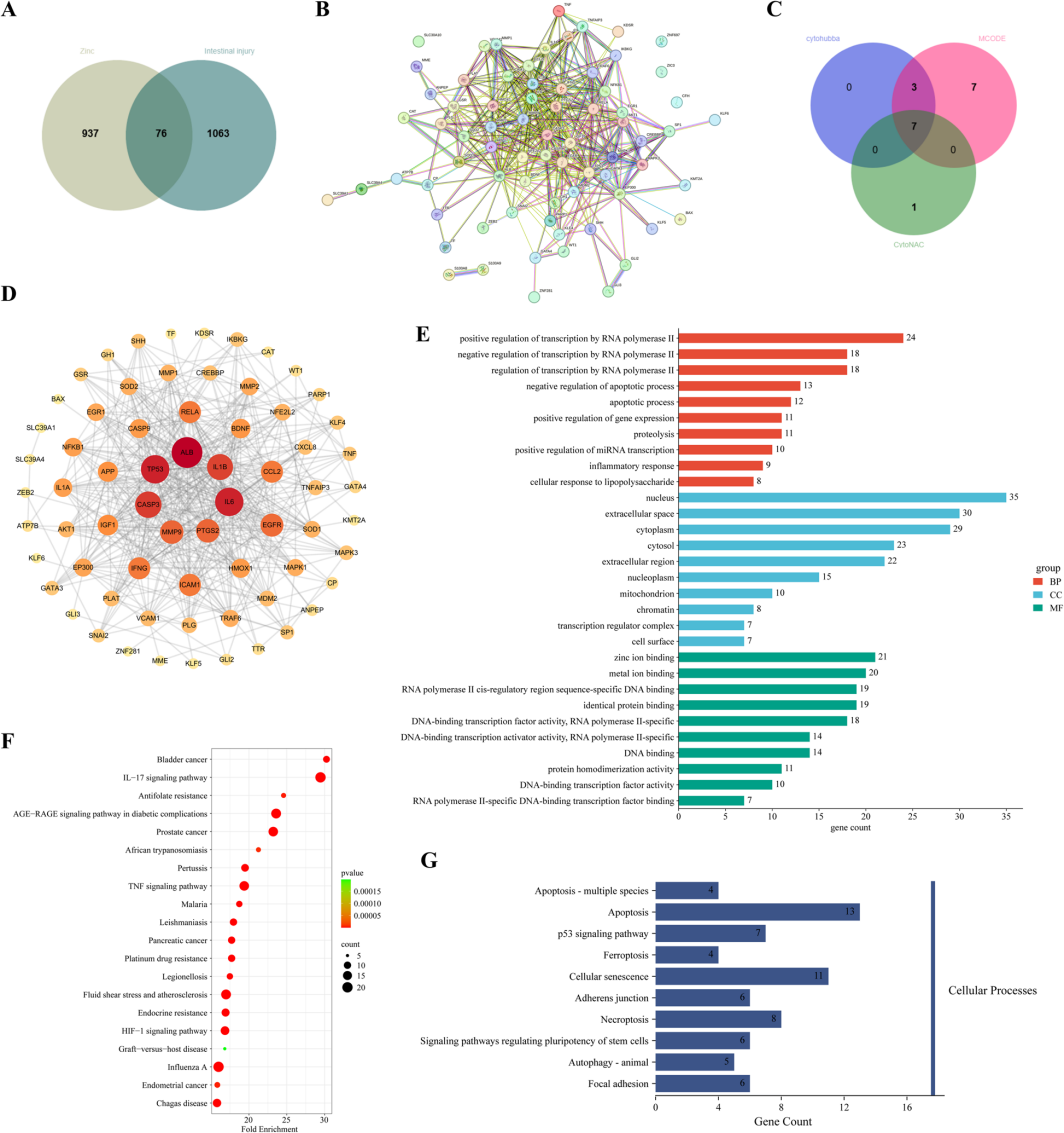
Identification of core targets in zinc-induced intestinal injury. **A** Overlapping targets from the intersection. **B** PPI network of potential targets. **C** Overlapping hub genes identified from multiple analytical methods. **D** The PPI network of 7 core targets and others. **E** Bar plot of GO enrichment analysis. **F** Top 20 enriched KEGG pathways. **G** Specific sub-pathways enriched in the KEGG Cellular Processes category

Network toxicology was employed to identify mechanisms underlying zinc-induced intestinal injury. Intersection of zinc- and intestinal injury-related genes yielded 79 overlapping targets (Fig. 1A). PPI network and Cytoscape plugins (cytohubba, MCODE, and CytoNCA) analysis identified seven hub genes: albumin (*ALB*), interleukin-6 (*IL-6*), tumor protein P53 (*TP53*), Caspase3, interleukin-1β (*IL-1β*), prostaglandin G/H synthase 2 (*PTGS2*), and matrix metalloproteinase 9 (*MMP9*) (Fig. 1B–D). GO analysis revealed 343 significant terms, primarily involving transcriptional regulation and apoptotic signaling (Fig. 1E). KEGG pathway analysis identified 135 significantly enriched pathways, many of which converged on inflammation and stress response, such as the IL-17, TNF, and hypoxia inducible factor-1 (HIF-1) signaling pathways (Fig. 1F). Notably, within the Cellular Processes category, four programmed cell death pathways were specifically enriched: apoptosis, ferroptosis, necroptosis, and autophagy (Fig. 1G).

### Ferroptosis is the predominant cell death pathway induced by zinc overload in IPEC-J2 cells

**Fig. 2:**
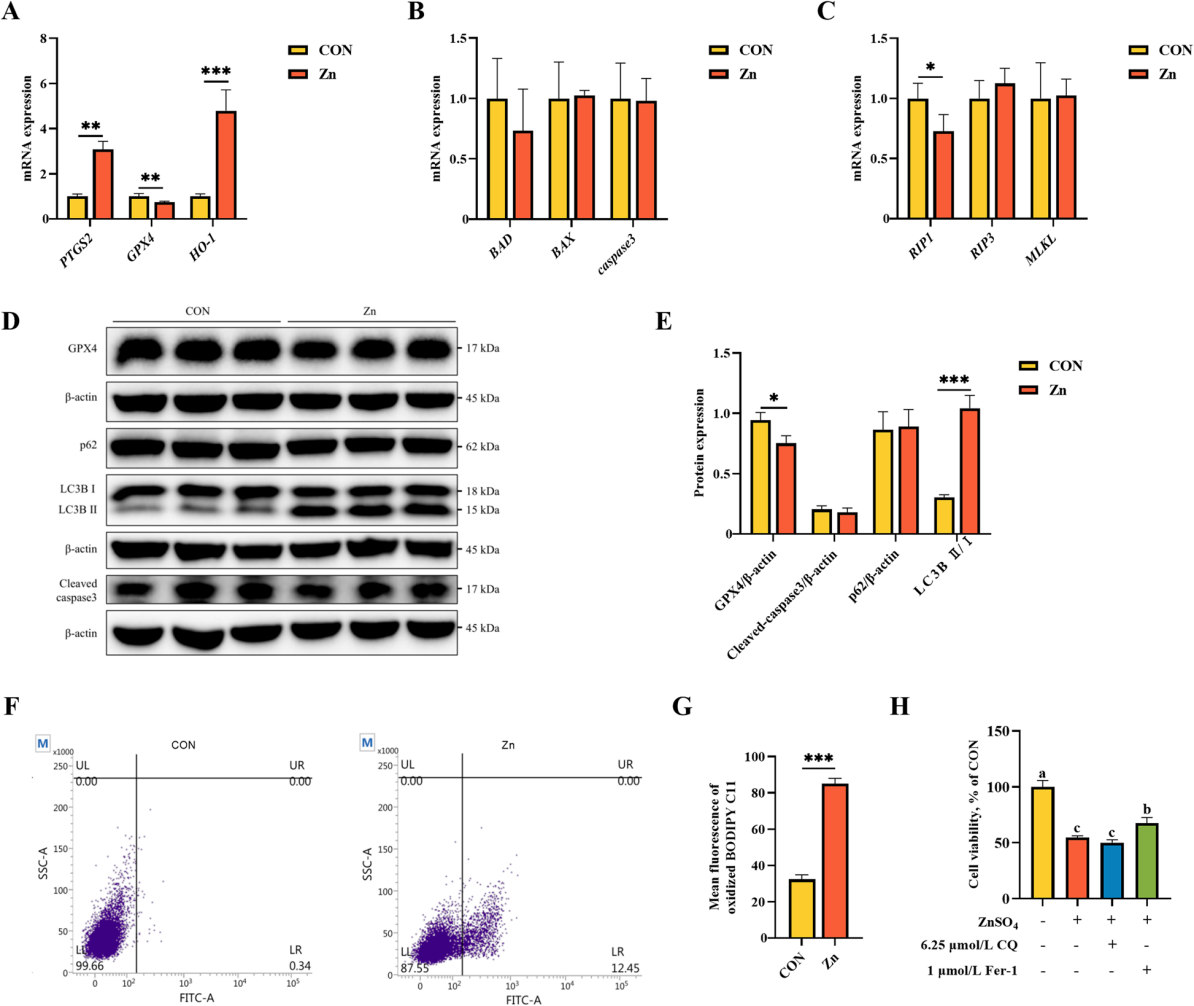
Zinc overload specifically induces ferroptosis in IPEC‑J2 cells. **A**–**C** The mRNA expressions of ferroptosis-, apoptosis-, and necroptosis-related genes (*n* = 4). **D** and **E** Protein levels of key markers for ferroptosis (GPX4), apoptosis (cleaved caspase 3), and autophagy (p62 and LC3B II/I) (*n* = 3). **F** and **G** Flow cytometry analysis and quantitative results of oxidized BODIPY C11 (*n* = 3). **H** Viability of zinc-overloaded IPEC-J2 cells treated with CQ and Fer-1 (*n* = 6). Data are expressed as mean ± SD. ^*^P < 0.05, ^**^P < 0.01, and ^***^P < 0.001. Bars with different letters indicate significant differences (*P* < 0.05)

To identify the predominant cell death pathway activated by zinc overload, key molecular markers associated with apoptosis, ferroptosis, necroptosis, and autophagy were systematically examined. Zinc overload significantly upregulated ferroptosis‑related genes *PTGS2* and *HO-1*, alongside a marked downregulation of *GPX4* (Fig. 2A). In contrast, the expression levels of key apoptotic regulators namely B-cell lymphoma 2-associated X protein (*BAX*), BCL2-associated agonist of cell death (*BAD*), and *caspase 3* remained unchanged (Fig. 2B). Similarly, the mRNA levels of the core necroptosis mediators, receptor-interacting serine/threonine-protein kinase 1 (*RIP1*), *RIP3* and mixed lineage kinase domain-like protein (*MLKL*) were assessed. Notably, while *RIP1* expression was reduced, *RIP3* and *MLKL* levels showed no significant alteration (Fig. 2C). At the protein level, zinc overload significantly downregulated ferroptosis marker GPX4 and upregulated autophagy marker LC3B II/I ratio, while leaving apoptosis marker cleaved caspase 3 unchanged (Fig. 2D and E). Direct measurement of lipid peroxidation using BODIPY™ 581/591 C11 probe showed that zinc overload significantly increased oxidized BODIPY C11 fluorescence (Fig. 2F and G). Moreover, the ferroptosis inhibitor Fer-1 effectively rescued cell viability, whereas the autophagy inhibitor CQ failed to provide protection (Fig. 2H).

### Screening identifies baicalein (BAI) and FIS as non-chelating polyphenols that protect intestinal barrier integrity under zinc overload

Primary screening of 22 polyphenols (5–30 μmol/L) identified four candidates, BAI, FIS, isorhamnetin (ISO), and 3,4-dihydroxybenzaldehyde (MDHB), that consistently protected IPEC-J2 cells from zinc-induced cytotoxicity without chelating zinc (Fig. S2).

**Fig. 3:**
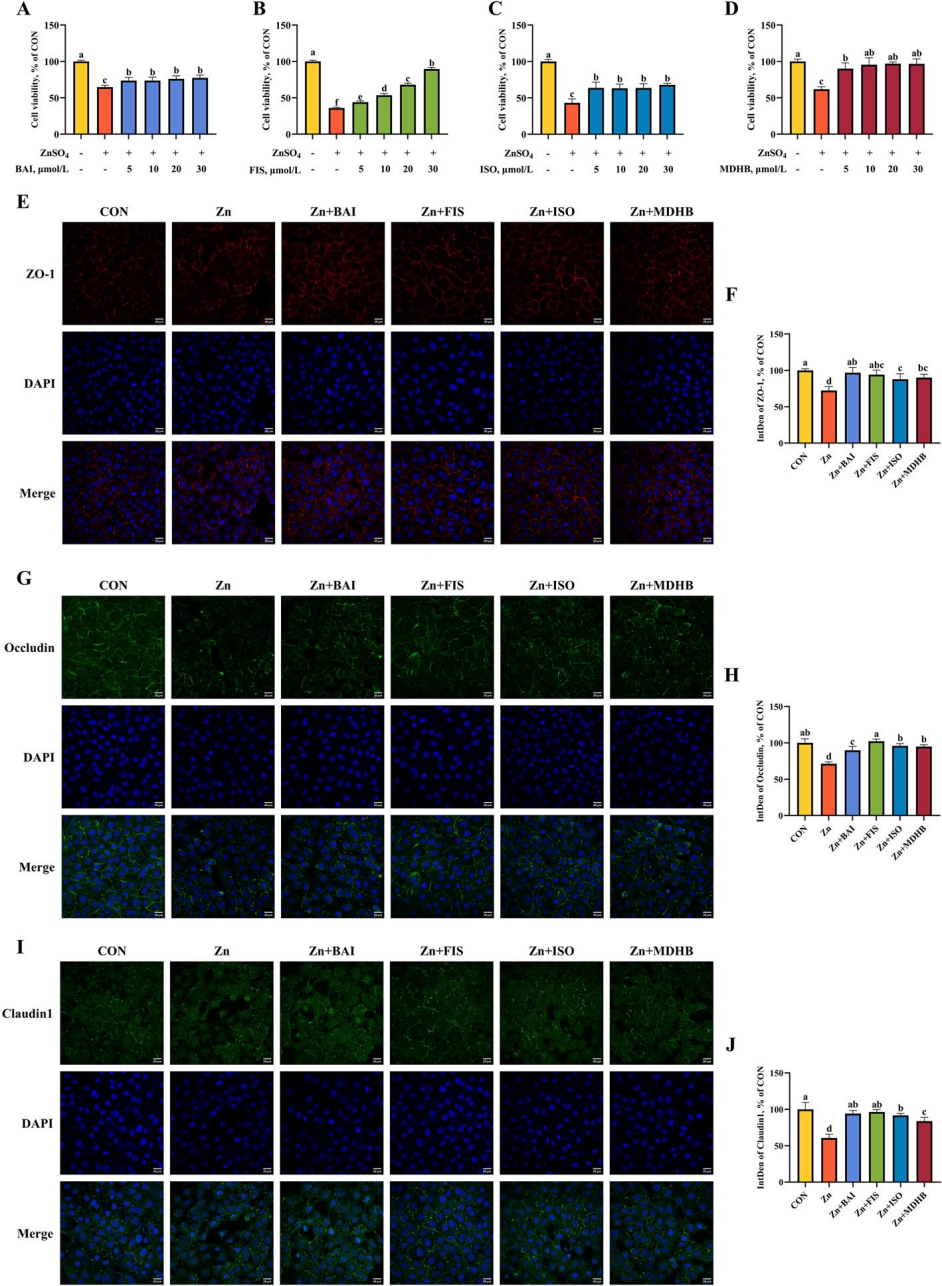
The selected polyphenols protect intestinal epithelial barrier integrity under zinc overload in IPEC-J2 cells. **A**–**D** Viability of zinc-overloaded IPEC-J2 cells treated with BAI, FIS, ISO, and MDHB at concentrations of 5, 10, 20, and 30 μmol/L (*n* = 4). **E** and **F** Representative immunofluorescence images and quantitative analysis of ZO-1 (red) (*n* = 5). **G** and **H** Representative immunofluorescence images and quantitative analysis of Occludin (green) (*n* = 5). **I** and **J** Representative immunofluorescence images and quantitative analysis of Claudin1 (green) (*n* = 5). Nuclei are stained with DAPI (blue). Scale bar, 20 μm. Data are expressed as mean ± SD. Bars with different letters indicate significant differences (*P* < 0.05)

Secondary screening assessed the barrier protective properties of these four candidates in zinc-overloaded IPEC-J2 cells. A dose–response assessment confirmed that 30 μmol/L was the most effective concentration for all four polyphenols in restoring cell viability (Fig. 3A–D), and this concentration was therefore employed in subsequent cell experiments. Immunofluorescence analysis demonstrated that all four compounds significantly alleviated the zinc-induced downregulation of the tight junction proteins ZO-1, Occludin, and Claudin1 (Fig. 3E–J). Quantitative analysis showed distinct restorative efficacies among the polyphenols, BAI was most potent in restoring ZO-1, FIS was most effective for Occludin, and both BAI and FIS exhibited superior efficacy in rescuing Claudin1 expression (Fig. 3F, H, and J).

### FIS exhibits superior antioxidant and anti-inflammatory activities over BAI

**Fig. 4:**
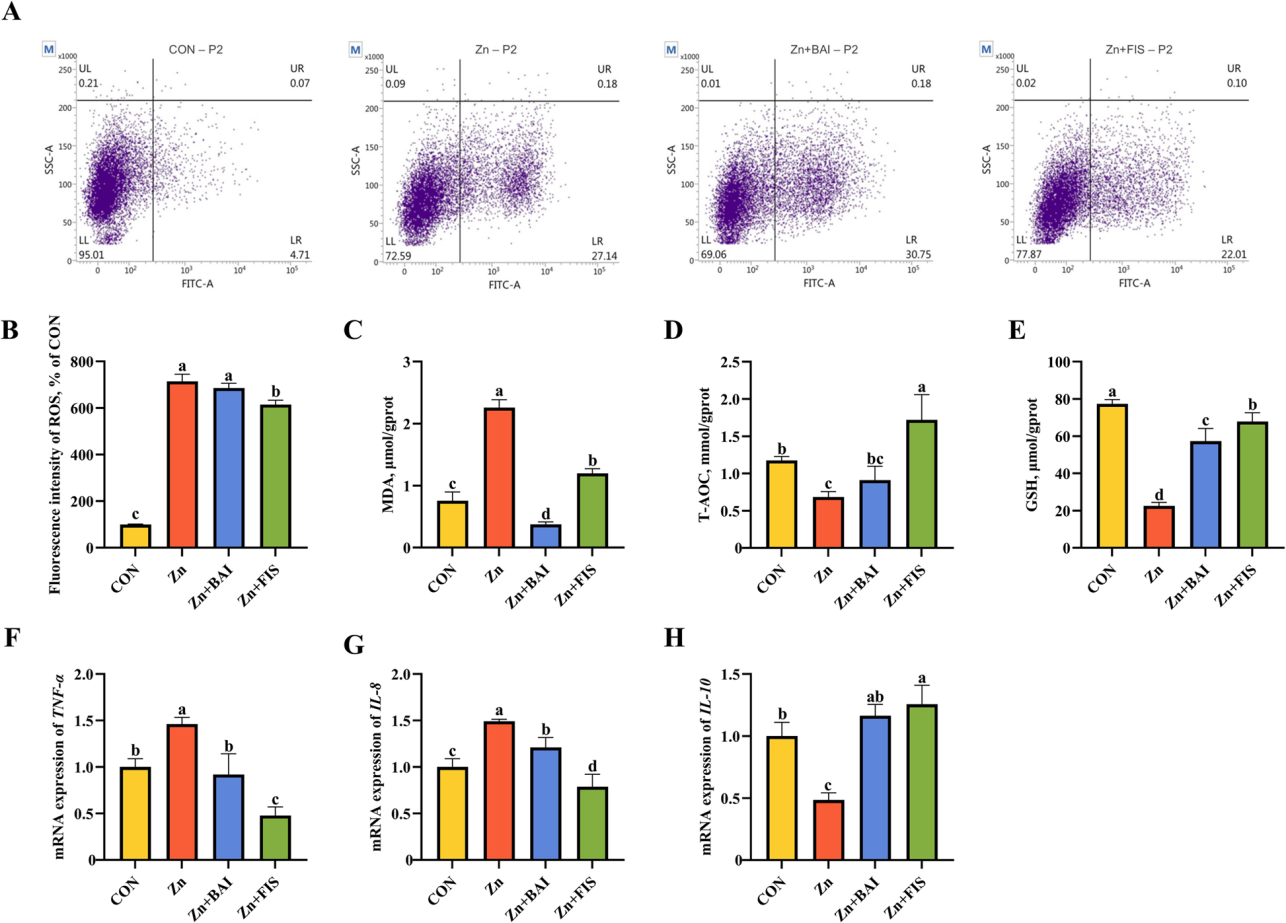
BAI and FIS alleviate oxidative stress and inflammation in zinc-overloaded IPEC-J2 cells. **A** and **B** Representative fluorescence images and quantitative analysis of ROS (*n* = 3). **C**–**E** MDA, T-AOC, and GSH levels (*n* = 3). **F**–**H** Relative mRNA expression of *TNF-α*, *IL-8*, and *IL-10* (*n* = 4). Data are expressed as mean ± SD. Bars with different letters indicate significant differences (*P* < 0.05)

FIS most potently suppressed zinc-induced ROS burst (Fig. 4A and B). Both BAI and FIS reduced the level of lipid peroxidation product MDA (Fig. 4C), while FIS alone enhanced T-AOC (Fig. 4D). Both polyphenols restored depleted GSH (Fig. 4E). Regarding inflammation, both suppressed TNF-α and rescued IL-10, while FIS additionally inhibited IL-8 upregulation (Fig. 4F–H).

### FIS inhibits zinc overload-induced ferroptosis by activating the Nrf2-GPX4 axis in IPEC-J2 cells

**Fig. 5:**
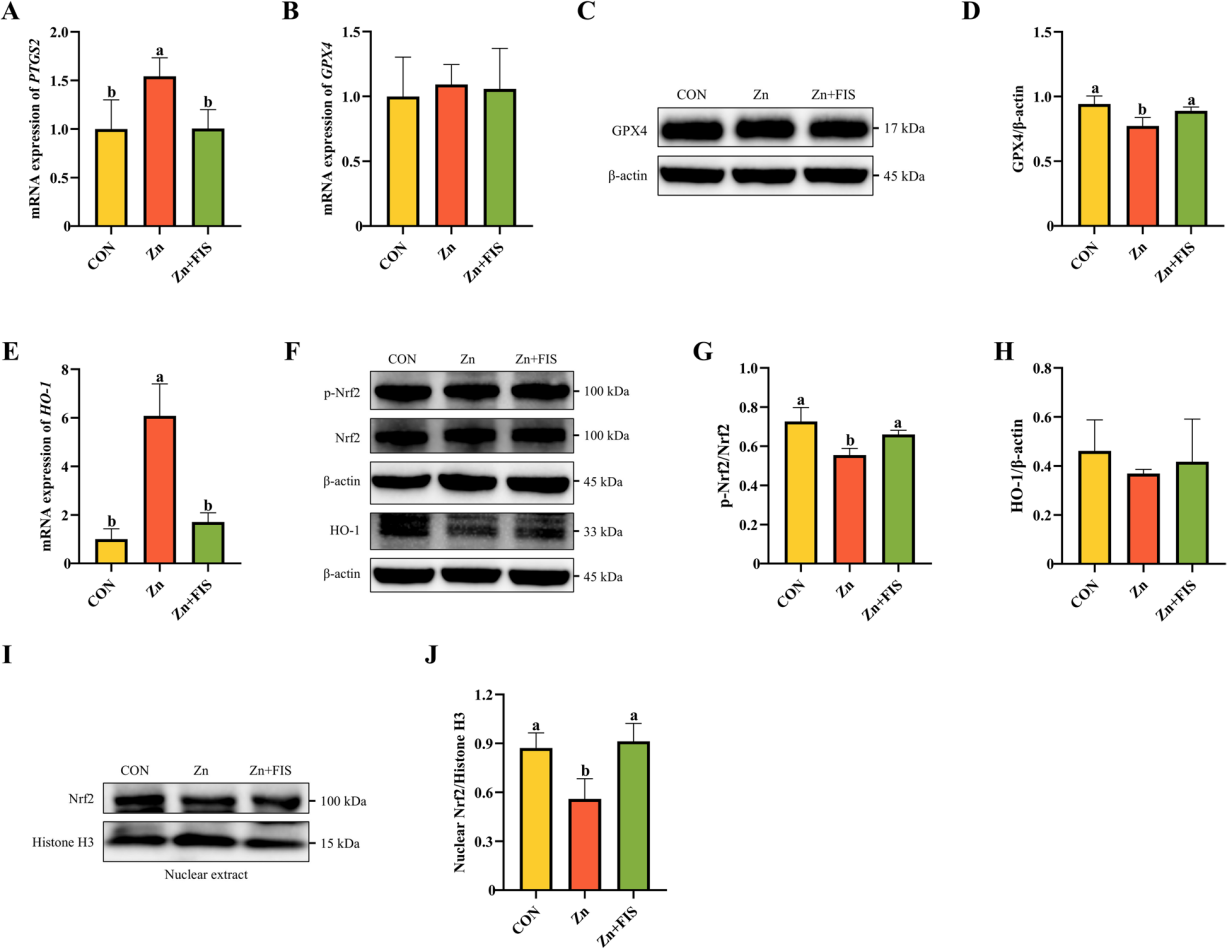
FIS inhibits zinc overload-induced ferroptosis by activating the Nrf2-GPX4 axis in IPEC‑J2 cells. **A** and **B** The mRNA expressions of the ferroptosis marker *PTGS2* and *GPX4* (*n* = 4). **C** and **D** Representative Western blotting band and quantification of GPX4 in whole-cell lysates (*n* = 3). **E** The mRNA expressions of *HO-1* (*n* = 4). **F**–**H** Representative Western blotting bands and quantification of p-Nrf2, Nrf2 and HO-1 in whole-cell lysates (*n* = 3). **I** and **J** Representative Western blotting bands and quantification of nuclear Nrf2 (*n* = 3). Data are expressed as mean ± SD. Bars with different letters indicate significant differences (*P* < 0.05)

FIS treatment significantly suppressed the zinc-induced upregulation of the ferroptosis marker gene *PTGS2* (Fig. 5A) and restored GPX4 expression at protein levels (Fig. 5B–D). To elucidate the upstream mechanism, the Nrf2 antioxidant pathway, a key regulator of GPX4 transcription, were examined. Zinc overload triggered a significant increase in the mRNA level of the Nrf2 target gene *HO‑1*, which was effectively mitigated by FIS treatment (Fig. 5E). FIS also restored the ratio of p‑Nrf2/Nrf2 and nuclear Nrf2, which was suppressed under zinc overload (Fig. 5F–J).

### FIS improves growth performance and intestinal morphology in zinc-overloaded piglets

**Fig. 6:**
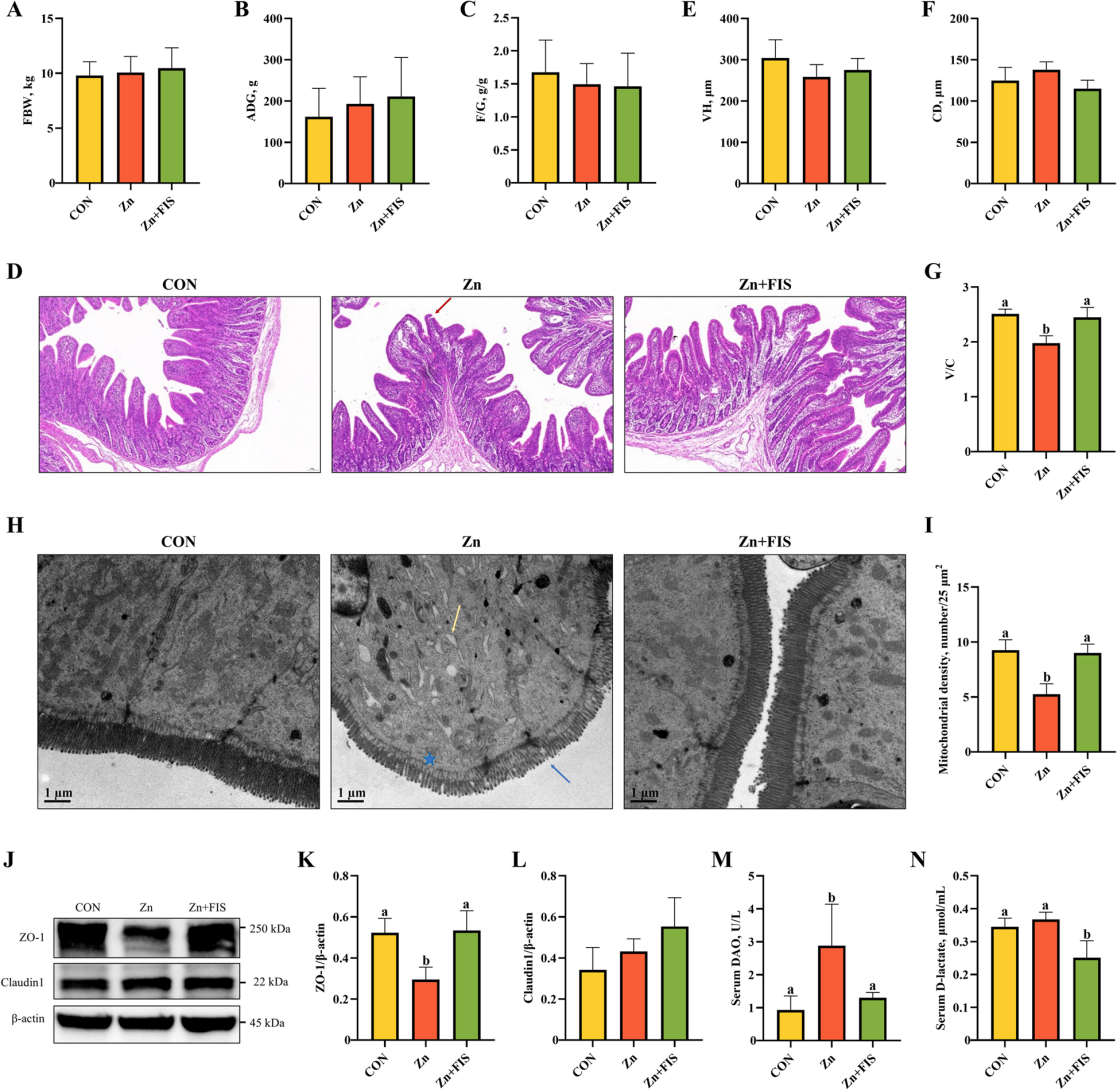
Effects of FIS on the growth performance and intestinal morphology of zinc-overloaded piglets. **A**–**C** Final body weight, ADG, and F/G of piglets (*n* = 10). **D** Representative H&E-stained images of the piglet jejunum. The red arrow indicates villus erosion. Scale bars: 100 μm. **E**–**G** VH, CD, and V/C ratio of jejunum (*n* = 3). **H** TEM images of jejunum. The blue arrow indicates shortened microvilli with diminished density, the blue star indicates mitochondria with cristae loss, and the yellow arrow indicates dilated endoplasmic reticulum. Scale bars: 1 μm. **I** Mitochondrial density of jejunum. **J** Representative Western blotting bands. **K** and **L** Quantification of ZO-1 and Claudin1 (*n* = 3). **M** Serum DAO activity (*n* = 3). **N** Serum D-lactate level (*n* = 3). Data are expressed as mean ± SD. Bars with different letters indicate significant differences (*P* < 0.05)

Dietary zinc overload promoted growth performance, increasing FBW and ADG while decreasing F/G. This effect was further enhanced by FIS supplementation (Fig. 6A–C). To assess whether FIS exerts zinc-chelating activity in vivo, Zn^2+^ concentrations in the serum and jejunum were measured. Zinc overload significantly increased the serum and jejunal Zn^2+^ levels, whereas FIS supplementation did not alter jejunal or serum Zn^2+^ levels (Fig. S3). Zinc overload caused villus erosion (red arrow, Fig. 6D), reduced villus height (VH)-to-crypt depth (CD) (V/C) ratio (Fig. 6E–G), and caused ultrastructural damage including shortened microvilli with diminished density (blue arrow), loss of mitochondrial cristae and density (blue star), and dilation of the endoplasmic reticulum (yellow arrow) (Fig. 6H and I). FIS supplementation reversed these structural impairments (Fig. 6D–I) and restored ZO-1 protein level, while further elevating Claudin1 without statistical significance (Fig. 6J–L). Regarding intestinal permeability, serum DAO activity, a direct marker of intestinal mucosal injury, was significantly increased by zinc overload. Although serum D‑lactate levels did not differ significantly between the control and zinc overload groups, FIS treatment significantly reduced both serum DAO and D‑lactate levels compared with the zinc overload group (Fig. 6M and N).

### FIS alleviates zinc overload-induced intestinal oxidative stress, ferroptosis, and inflammation in piglets

**Fig. 7:**
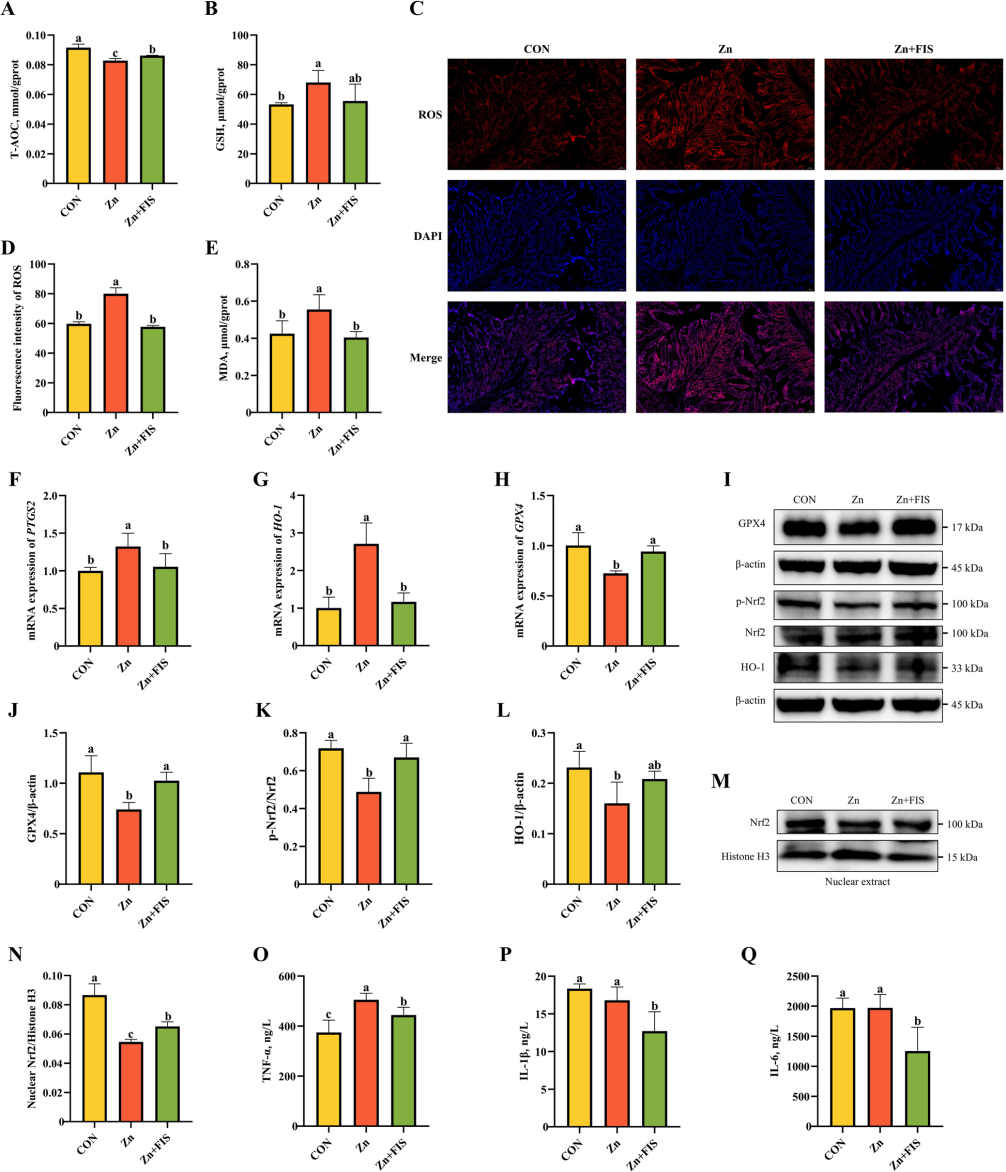
FIS alleviates zinc overload-induced oxidative stress and inflammation in piglets. **A** and **B** Levels of T-AOC and GSH in the jejunum of piglets (*n* = 4). **C** and **D** Representative immunofluorescence images and quantitative analysis of ROS (red) in frozen sections in the jejunum of piglets (*n* = 4). Nuclei are stained with DAPI (blue). Scale bar: 100 μm. **E** Level of MDA in the jejunum of piglets (*n* = 4). **F**–**H** The mRNA expressions of ferroptosis-related genes *PTGS2*, *HO-1*, and *GPX4* (*n* = 3). **I** Representative Western blotting bands in whole-cell lysates. **J**–**L** Quantification of GPX4, p-Nrf2/Nrf2 ratio, and HO-1 (*n* = 3). **M** Representative Western blotting bands in nuclear extracts. **N** Quantification of nuclear Nrf2 (*n* = 3). **O**–**Q** Levels of TNF-α, IL-1β, and IL-6 in the jejunum of piglets (*n* = 4). Data are expressed as mean ± SD. Bars with different letters indicate significant differences (*P* < 0.05)

Zinc overload induced significant oxidative stress, evidenced by increased levels of GSH, ROS, and MDA, along with a decreased level of T-AOC. Treatment with FIS effectively attenuated the increase in ROS and MDA as well as the decrease in T-AOC (Fig. 7A–E). Zinc overload significantly upregulated *PTGS2* and *HO-1* mRNA, while downregulating *GPX4* mRNA. FIS supplementation effectively reversed all these alterations (Fig. 7F–H). Consistently, zinc overload reduced the protein levels of GPX4, p-Nrf2/Nrf2 ratio, nuclear Nrf2 and HO-1, all of which were significantly restored by FIS treatment (Fig. 7I–N). In terms of inflammation, zinc overload specifically increased TNF-α level, which was reversed by FIS (Fig. 7O). Although zinc overload did not significantly alter IL-1β or IL-6 levels, FIS supplementation further reduced the concentrations of both cytokines (Fig. 7P and Q).

### FIS modulates the gut microbiota in zinc-overloaded piglets

**Fig. 8:**
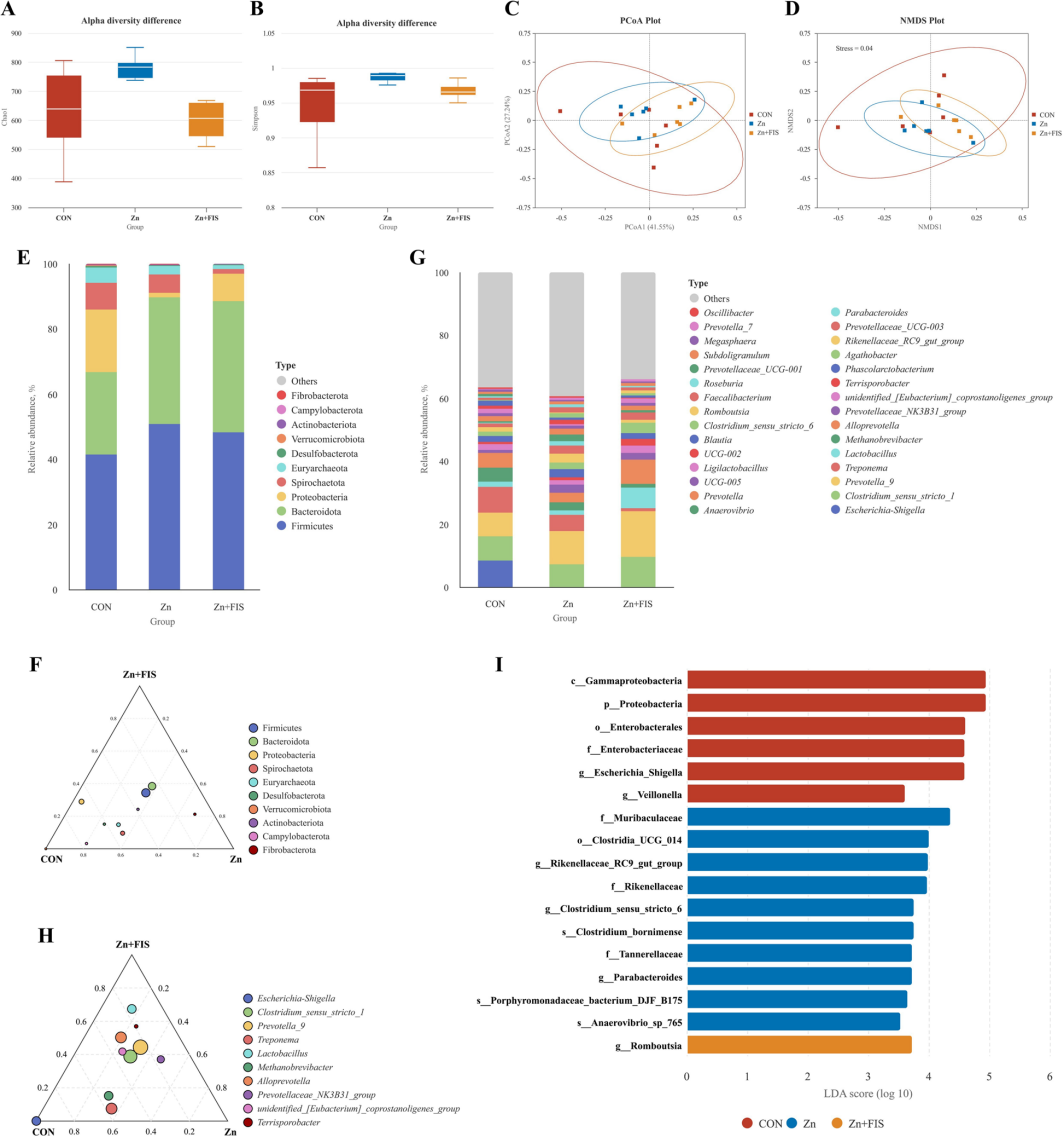
FIS remodels the cecal microbial community structure in zinc-overloaded piglets. **A** and **B** Alpha diversity indices (Chao1 for richness, Simpson for diversity) of the cecal microbiota across groups. **C** and **D** Beta diversity visualized by PCoA (weighted UniFrac distance) and NMDS (weighted UniFrac distance). **E** Relative abundance of the cecal microbiota at the phylum level (top 10 taxa shown). **F** Ternary plot at the phylum level. **G** Relative abundance of the cecal microbiota at the genus level (top 30 taxa shown). **H** Ternary plot at the genus level. **I** LEfSe analysis (LDA score > 3.5, *P* < 0.05)

Alpha diversity (Chao1, Shannon) remained unchanged among groups (Fig. 8A and B), while beta diversity (PCoA, NMDS) revealed distinct microbial communities following zinc overload and FIS treatment (Fig. 8C and D). At the phylum level, the microbiota was predominantly composed of Firmicutes, Bacteroidota, and Proteobacteria (Fig. 8E). Ternary plot analysis showed that the CON group was enriched with Verrucomicrobiota, the Zn group was characterized by an increase in rare phyla such as Fibrobacterota, while the Zn + FIS group exhibited a distinct aggregation of the dominant phyla Firmicutes and Bacteroidota (Fig. 8F). At the genus level, the CON group was dominated by *Escherichia-Shigella*. The Zn group showed a significant enrichment of *Prevotellaceae_NK3B31_group*, whereas the Zn + FIS group exhibited a distinct microbial structure, characterized by the aggregation of beneficial genera including *Lactobacillus*, *Terrisporobacter*, *Alloprevotella*, *Clostridium_sensu_stricto_1*, and *Prevotella_9* (Fig. 8G and H). Supporting these observations, Kruskal–Wallis tests indicated that the relative abundance of the enteropathogen *Escherichia-Shigella* was significantly higher in the CON group. In the Zn group, *Rikenellaceae_RC9_gut_group, Parabacteroides* and *Clostridium_sensu_stricto_6* were significantly elevated. *Romboutsia* was uniquely enriched in the Zn + FIS group (Fig. S4), indicating that FIS could directionally enrich beneficial cecal microbes. To pinpoint microbial biomarkers characteristic of each treatment, LEfSe analysis was performed with an LDA score threshold of > 3.5. The CON group was associated with Proteobacteria (Gammaproteobacteria, Enterobacteriaceae, *Enterobacterales*, *Escherichia-Shigella*) and *Veillonella*. The Zn group was characterized by 10 discriminatory taxa, including Muribaculaceae, Clostridia_UCG_014, Rikenellaceae (*Rikenellaceae_RC9_gut_group*), *Clostridium_sensu_stricto_6* (*Clostridium_bornimense*), Tannerellaceae (*Parabacteroides*), *Porphyromonadaceae_bacterium_DJF B175*, and *Anaerovibrio_sp_765*. Notably, *Romboutsia* was identified as the sole significant biomarker for the Zn + FIS group (Fig. 8I).

### FIS-induced gut microbiota modeling link to improved host phenotypes and ferroptosis defense

**Fig. 9:**
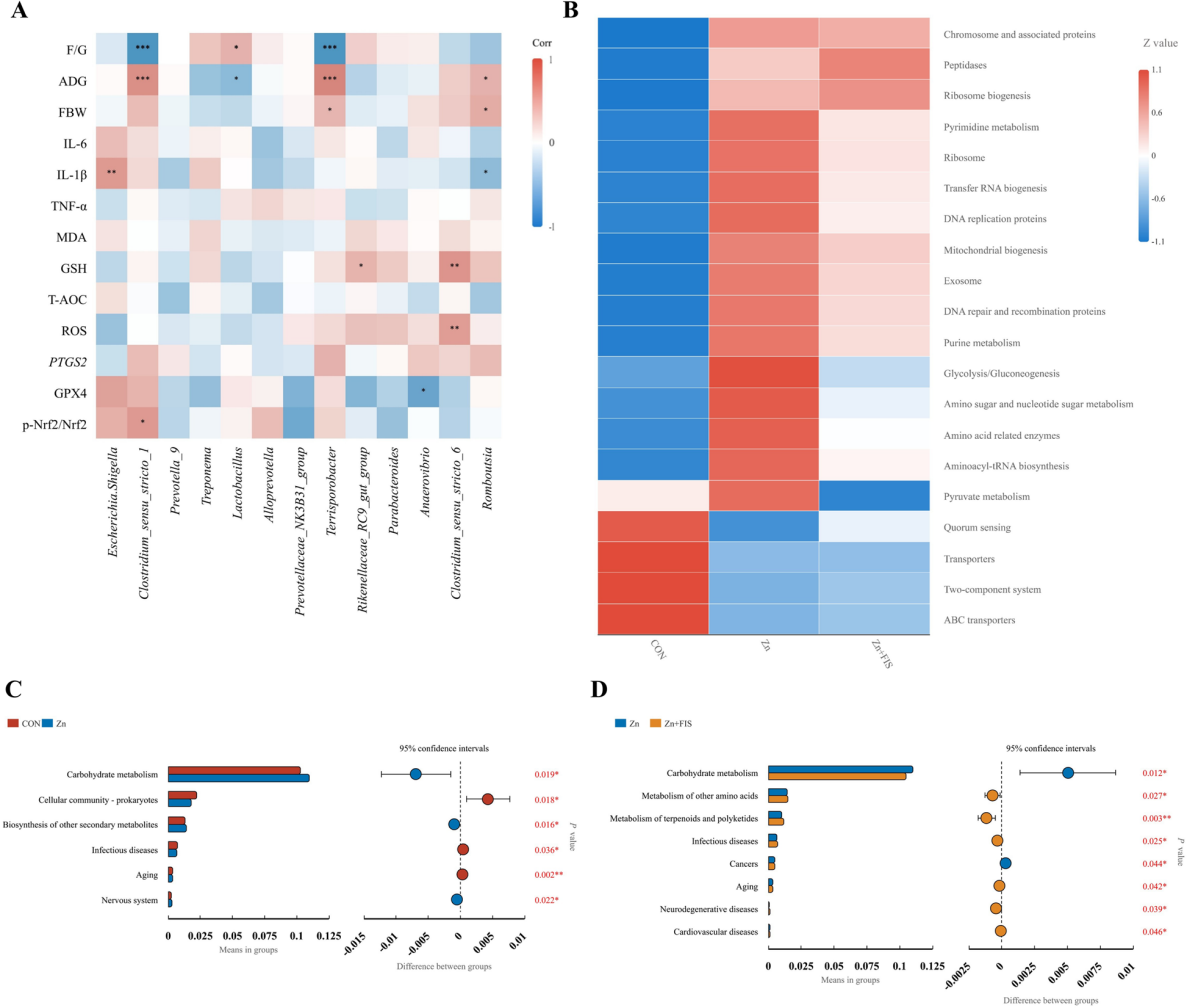
FIS-induced gut microbiota modeling link to improved host phenotypes and ferroptosis defense. **A** Spearman correlation heatmap between key microbial genera at genus level and host phenotypic parameters red indicates a positive correlation and blue indicates a negative correlation. **B** Heatmap of predicted microbial metabolic pathway abundances. The color gradient indicates relative abundance (red, high; blue, low). **C** and **D** Bar plots showing differentially abundant KEGG pathways (level 2) for the comparisons CON vs. Zn and Zn vs. Zn + FIS. Only pathways with significant differences (*P* < 0.05) are displayed. ^*^P < 0.05, ^**^P < 0.01, and ^***^P < 0.001

Spearman correlation analysis revealed that beneficial genera such as *Romboutsia* (the biomarker for the Zn + FIS group) were positively correlated with ADG and FBW, and negatively correlated with IL-1β. *Clostridium_sensu_stricto_1* correlated positively with ADG and negatively with F/G, while *Escherichia-Shigella* (the biomarker for the CON group) correlated positively with IL-1β. Specific correlations with oxidative stress markers were also observed; for instance, *Rikenellaceae_RC9_gut_group* was positively correlated with GSH level. Notably, the associations between specific genera and ferroptosis markers provided new insights into the anti-ferroptotic mechanism of FIS. *Clostridium_sensu_stricto_1* was significantly positively correlated with the p-Nrf2/Nrf2 ratio. *Anaerovibrio*, identified by LEfSe as a biomarker for the zinc overload group, was significantly negatively correlated with GPX4 protein expression (Fig. 9A). Functional profiling revealed distinct alterations across groups (Fig. 9B). Compared to the CON group, the Zn group exhibited upregulated carbohydrate metabolism and downregulated pathways for cellular community (Fig. 9C). In contrast, FIS supplementation reversed these changes, reducing carbohydrate metabolism while enriching pathways for amino acid metabolism and metabolism of terpenoids and polyketides (Fig. 9D).

## Discussion

This study systematically elucidated the mechanisms underlying high-dose zinc-induced intestinal injury through integrated network toxicology, in vitro screening, and in vivo functional evaluation. FIS was identified as a non-chelating polyphenol that alleviates zinc toxicity by activating the Nrf2-GPX4 axis to inhibit ferroptosis and remodeling gut microbiota, while preserving zinc's growth benefits.

Network toxicology predicted that zinc overload may activate multiple cell death pathways, but experimental validation in IPEC-J2 cells confirmed ferroptosis as the predominant mechanism, evidenced by selective alterations in PTGS2 and GPX4 without changes in apoptosis, necroptosis, or autophagy markers. This apparent discrepancy may be explained by the fact that network toxicology predictions integrate findings from diverse experimental conditions and sample types, whereas under our specific zinc overload setting, the activation of apoptosis requires higher zinc concentrations or longer exposure durations than those used here [24, 25]. Furthermore, although previous studies have reported zinc-induced ferroptosis in other cell types [11, 12], this study systematically confirms this mechanism in porcine intestinal epithelial cells and a weaned piglet model.

High doses of ZnO are commonly employed to manage post-weaning diarrhea [26, 27]. In this study, 1,600 mg/kg ZnO yielded the expected growth-promoting effects but concurrently induced oxidative stress, ferroptosis, and microbial dysbiosis, providing new evidence for the double-edged sword effect of high-dose zinc [3, 28]. Although its antimicrobial and astringent properties likely contribute to the initial control of digestive disturbances [29], this benefit comes at the cost of disrupting intestinal homeostasis and microbial balance. To address these challenges while preserving zinc's growth benefits, a targeted screening strategy was developed to identify non-chelating polyphenols. From 22 polyphenols, FIS emerged as the most promising candidate based on its ability to alleviate zinc-induced cytotoxicity independent of zinc chelation, protect tight junction proteins, and exhibit superior antioxidant and anti-inflammatory activities.

Mechanistically, FIS restored zinc-suppressed Nrf2 phosphorylation and HO-1 expression, activating the Nrf2-GPX4 axis to reverse PTGS2 upregulation, thereby inhibiting ferroptosis. In piglets, FIS preserved growth performance while ameliorating jejunal villus erosion, ultrastructural damage, and tight junction disruption, as well as alleviating oxidative stress and inflammatory responses, consistent with in vitro findings.

Beyond direct protection, this study uncovered ecological risks of ZnO supplementation. While confirming enteropathogens like *Escherichia-Shigella* [8], ZnO enriched resistance-associated taxa including *Parabacteroides* and Tannerellaceae, known reservoirs of antibiotic resistance genes [30, 31]. This aligns with evidence that heavy metals, including zinc, may promote resistance gene dissemination through co-selection [32], implying that high-dose ZnO may pose a potential long-term risk to animal and public health [33, 34]. Functional prediction further revealed upregulated carbohydrate metabolic pathways in the ZnO group, corresponding to enrichment of carbohydrate metabolism-associated bacteria such as Muribaculaceae and *Clostridium_bornimense*.

In contrast, FIS reshaped the microbiota rather than merely reversing ZnO-induced changes. It enriched beneficial taxa (*Romboutsia*, *Clostridium_sensu_stricto_1*), while avoiding the enrichment of resistance-associated taxa like *Parabacteroides*. *Romboutsia* abundance correlated positively with improved growth performance and lower inflammatory level, implying the microbiota mediates host benefits [35, 36]. Critically, direct links between specific taxa and ferroptosis defense were identified. *Clostridium_sensu_stricto_1* (an SCFA-producing genus) correlated positively with both growth performance and the p-Nrf2/Nrf2 ratio, indicating FIS may activate Nrf2 through metabolites like butyrate [37] while concurrently promoting host growth. This relationship may be bidirectional, as it is also plausible that Nrf2 activation by FIS directly or indirectly contributes to the observed gut microbiota changes, as Nrf2 signaling has been shown to favorably alter microbial composition by reducing oxidative stress and improving the intestinal microenvironment [38, 39]. Additionally, *Anaerovibrio* (a lipolytic genus enriched by zinc) correlated negatively with GPX4 protein expression. As a lipolytic genus, *Anaerovibrio* may hydrolyze triglycerides to release free fatty acids, potential substrates for lipid peroxidation, thereby exacerbating ferroptotic damage when GPX4 is inhibited [40]. Functional prediction further supported these findings, showing FIS enrichment of amino acid and terpenoid/polyketide metabolism pathways, which may provide glutathione precursors and natural Nrf2 agonists, respectively [41, 42]. Collectively, these findings elucidate that FIS-induced microbiota remodeling synergistically inhibits ferroptosis through dual modulation of beneficial and harmful bacteria.

In summary, FIS remodels the gut microbiota toward a beneficial community enriched with *Romboutsia* and *Clostridium_sensu_stricto_1*, the latter positively correlated with Nrf2 activation, while suppressing zinc-enriched *Anaerovibrio* negatively correlated with GPX4. These microbial shifts, coupled with direct activation of the Nrf2-GPX4 axis, synergistically inhibit ferroptosis and improve intestinal homeostasis. From a practical perspective, the ability of FIS to preserve the growth-promoting benefits of high-dose zinc while mitigating its intestinal toxicity and ecological risks positions it as a promising feed additive for sustainable swine production. Although this study establishes the potential of FIS in mitigating zinc toxicity, further research is required to identify its precise molecular targets and establish causal roles of specific microbiota through fecal transplantation studies.

## Conclusion

This study demonstrates that FIS, a non-chelating polyphenol, alleviates high-dose zinc-induced intestinal injury in weaned piglets by activating the Nrf2-GPX4 axis to inhibit ferroptosis, an effect that was associated with concurrent gut microbiota remodeling. By integrating direct cytoprotection with indirect ecological modulation, FIS preserves the growth-promoting benefits of zinc while mitigating its toxicity, positioning it as a promising feed additive for sustainable animal production.

## Supplementary Information

Additional file 1: Table S1. The complete list of 22 polyphenols. Table S2. The detailed composition and nutrient levels of the basal diet. Table S3. The primers sequence for RT-qPCR. Fig. S1. The chemical structures of 22 polyphenols. Fig. S2. Screening for non-chelating polyphenols alleviating zinc-induced cytotoxicity in IPEC-J2 cells. Fig. S3. Effects of FIS on Zn^2+^ concentrations in zinc-overloaded piglets. Fig. S4. Kruskal-Wallis test bar plot of differential cecal microbiota at the genus level. Fig. S5. Full unedited PVDF membranes for Fig. 2D. Fig. S6. Full unedited PVDF membranes for Fig. 5C. Fig. S7. Full unedited PVDF membranes for Fig. 5F. Fig. S8. Full unedited PVDF membranes for Fig. 5I. Fig. S9. Full unedited PVDF membranes for Fig. 6J. Fig. S10. Full unedited PVDF membranes for Fig. 7I. Fig. S11. Full unedited PVDF membranes for Fig. 7M.

## Data Availability

The datasets used and analyzed during this study are available from the corresponding author upon reasonable request.

## Abbreviations

ADG: Average daily gain
BAD: BCL2-associated agonist of cell death
BAX: B-cell lymphoma 2-associated X protein
BAI: Baicalein
CCK-8: Cell counting kit-8
CD: Crypt depth
CQ: Chloroquine
DAO: Diamine oxidase
FBW: Final body weight
F/G: Feed-to-gain ratio
Fer-1: Ferrostatin-1
FIS: Fisetin
GSH: Glutathione
GPX4: Glutathione peroxidase 4
H&E: Hematoxylin and eosin
HO-1: Heme oxygenase-1
HIF-1: Hypoxia inducible factor-1
HRP: Horseradish peroxidase
IL: Interleukin
ISO: Isorhamnetin
LC3B: Microtubule‑associated protein 1 light chain 3B
LEfSe: Linear discriminant analysis effect size
MDA: Malondialdehyde
MDHB: 3,4-Dihydroxybenzaldehyde
MLKL: Mixed lineage kinase domain-like protein
NMDS: Nonmetric multidimensional scaling
Nrf2: Nuclear factor erythroid 2-related factor 2
p62: Sequestosome‑1
PBS: Phosphate-buffered saline
PCoA: Principal coordinate analysis
PVDF: Polyvinylidene fluoride
RIP1: Receptor-interacting serine/threonine-protein kinase 1
ROS: Reactive oxygen species
SDS-PAGE: Sodium dodecyl sulfate–polyacrylamide gel electrophoresis
T-AOC: Total antioxidant capacity
TEM: Transmission electron microscopy
TNF-α: Tumor necrosis factor-alpha
VH: Villus height
V/C: VH/CD
ZO-1: Zonula occludens-1
ZnO: Zinc oxide
