# Supplementary material for: Fisetin alleviates zinc overload-induced intestinal injury by inhibiting ferroptosis and remodeling gut microbiota in weaned piglets
Source: J Anim Sci Biotechnol. 2026 Aug 9;17:161. doi: 10.1186/s40104-026-01481-0 (PMC13453087; doi:10.1186/s40104-026-01481-0)
Supplement: Supplementary file 1 [file 40104_2026_1481_MOESM1_ESM.docx]

**Supplementary Information**

**Supplementary Table 1.** The complete list of 22 polyphenols.

| Polyphenols | Classes | Selection criteria （references） |
| --- | --- | --- |
| Proanthocyanidins (PC) | Flavonoids | Metal ion chelation [1] |
| Protocatechuic acid (PA) | Phenolic acids | Antioxidant, anti-inflammatory, alleviates cadmium toxicity [2] |
| Protocatechualdehyde (PCA) | Phenolic acids | Antioxidant, anti-inflammatory metabolite [3] |
| Liquiritin (LIQ) | Flavonoids | p38 MAPK/p53 pathway modulation, Antioxidant, anti-inflammatory [4] |
| 4-Hydroxyphenylacetic acid (4-HPAA) | Others | Antioxidant [5] |
| Neochlorogenic acid (NCGA) | Phenolic acids | Anti-inflammatory, upregulates HO-1 expression [6] |
| Isorhapontigenin (ISO) | Stilbenes | anti- inflammatory, antitumour [7] |
| Baicalein (BAI) | Flavonoids | Repairs lead induced damage, increase the abundance of beneficial gut microbiota [8] |
| Isoquercetin (ISOQ) | Flavonoids | Activates the ERK1/2-Nrf2 pathway [9] |
| Methyl-3,4-dihydroxybenzoate (MDHB) | Phenolic acids | Antioxidant, Nrf2 pathway [10] |
| Coixol (6-MBOA) | Others | Antioxidant, anti-inflammatory, neuroprotective [11] |
| Bergenin (BER) | Others | Anti-inflammatory, NLRP3 inflammasome activation [12] |
| Resveratrol (RES) | Stilbenes | Antioxidant, anti-inflammatory [13] |
| Myricitrin (MYR) | Flavonoids | Antioxidant, anti-inflammatory, antifibrotic [14] |
| Arbutin (ARB) | Others | Anti-inflammatory [15] |
| Punicalagin (PCG) | Others | Anti-colitic, modulates gut microbiota [16] |
| Quercetin (QUE) | Flavonoids | Antioxidant, anti-inflammatory, and anti-fibrotic [17] |
| Chlorogenic acid (CGA) | Phenolic acids | Anti-inflammatory, modulates gut microbiota [18] |
| Fisetin (FIS) | Flavonoids | Anti-inflammatory; mitigates arsenic-induced hepatic damage [19] |
| Veratric acid (VA) | Phenolic acids | Anti-inflammatory activity, PI3K/Akt/NF-kappa B signaling pathway [20] |
| Rosmarinic acid (RA) | Phenolic acids | Against copper-induced oxidative stress [21] |
| Epigallocatechin gallate (EGCG) | Flavonoids | anti-oxidant, anti-inflammatory, and metal chelation effects [22] |

**Supplementary Table 2.** The detailed composition and nutrient levels of the basal diet (as-fed basis, %).

| Ingredients | Content | Nutrient levels^2^ | Content |
| --- | --- | --- | --- |
| Corn | 50.00 | DE (MJ/kg) | 14.18 |
| Wheat flour | 9.00 | CP | 18.15 |
| Fermented soybean meal | 6.00 | EE | 5.09 |
| Extruded full-fat soybean | 2.00 | Calcium | 0.73 |
| Soybean meal | 2.00 | Phosphorus | 0.62 |
| Fish meal | 4.00 | Lysine | 1.40 |
| Cheese powder | 10.00 | Methionine | 0.51 |
| Whey powder | 10.00 | Threonine | 0.85 |
| Sucrose | 3.00 | Tryptophan | 0.23 |
| Calcium formate | 1.00 |  |  |
| Dicalcium phosphate | 0.80 |  |  |
| Salt | 0.30 |  |  |
| Lysine | 0.45 |  |  |
| Methionine | 0.15 |  |  |
| Threonine | 0.20 |  |  |
| Tryptophan | 0.05 |  |  |
| Choline chloride (60%) | 0.05 |  |  |
| Premix^1^ | 1.00 |  |  |
| Total | 100.00 |  |  |

DE = Digestible energy; CP = Crude protein; EE = Crude fat.

^1^Premix is provided per kg diet as follows: vitamin A, 6000 IU; vitamin D₃, 2200 IU; vitamin E, 20 IU; vitamin B₂, 5 mg; vitamin B₁₂, 0.021 mg; biotin, 0.1 mg; pantothenic acid, 10 mg; niacin, 15 mg; iron, 100 mg; copper, 6 mg; manganese, 10 mg; iodine, 0.30 mg; selenium, 0.30 mg; cobalt, 1 mg.

^2^DE was calculated value, while the others were analyzed values.

**Supplementary Table 3.** The primers sequence for RT-qPCR

| Gene name | Primer sequence（5'-3'） | Length（bp） |
| --- | --- | --- |
| *TNF-α* | F: TCCAATGGCAGAGTGGGTATG  R: AGCTGGTTGTCTTTCAGCTTCAC | 67 |
| *IL-8* | F: GACCCCAAGGAAAAGTGGGT  R: TGACCAGCACAGGAATGAGG | 186 |
| *IL-10* | F: CCTGACTGCCTCCCACTTTC  R: GGGCTCCCTAGTTTCTCTTCCT | 94 |
| *BAX* | F: CTACCAAGAAGTTGAGCGAGTG  R: CCAGTTGAAGTTGCCGTCAG | 158 |
| *BAD* | F: TCGGAAGAAGGCAGTGTGAC  R: AAGGACTCTGGCGGCGTTA | 207 |
| *Caspase3* | F: GGAATGGCATGTCGATCTGGT  R: ACTGTCCGTCTCAATCCCAC | 351 |
| *MLKL* | F: TCTCGCTGCTGCTTCA  R: CTCGCTTGTCTTCCTCTG | 105 |
| *RIP1* | F: ACATCCTGTACGGCAACTCT  R: CGGGTCCAGGTGTTTATCC | 175 |
| *RIP3* | F: CTTGTTGTCTGTCCGTGAGC  R: GAGGAGGTTGGGCTGTTGA | 238 |
| *PTGS2* | F: TCTTCCTCCTGTGCCTGATG  R: GCTGGTCCTCGCTTCTGAT | 204 |
| *GPX4* | F: CTGTTCCGCCTGCTGAA  R: ACCTCCGTCTTGCCTCAT | 218 |
| *β-actin* | F: TGCGGGACATCAAGGAGAAG  R: AGTTGAAGGTGGTCTCGTGG | 216 |

**Supplementary Figure 1.** The chemical structures of 22 polyphenols.

**Supplementary Results: Primary screening of 22 polyphenols**

All 22 polyphenols (listed in Supplementary Table S1) were evaluated in parallel for their ability to alleviate zinc-induced cytotoxicity in IPEC-J2 cells and their potential zinc-chelating activity. Cell viability was assessed after co-treatment with zinc and each polyphenol at four concentrations (5, 10, 20, and 30 μmol/L). Intracellular Zn²⁺ levels were measured concurrently to identify compounds that functioned primarily as zinc chelators.

At 5 μmol/L, the top eight polyphenols that most effectively improved cell viability were BAI, FIS, ISO, RES, RA, QUE, MDHB and CGA (Fig. S2A). When the concentration was increased to 10 μmol/L, the top performers were FIS, MDHB, ISO, RES, BAI, 6-MBOA, QUE, and MYR (Fig. S2B). As the evaluation continued at 20 μmol/L, the top eight were FIS, MDHB, PCG, PCA, ISO, BAI, 6-MBOA, and RES (Fig. S2C), and at 30 μmol/L, the most effective were FIS, PCA, MDHB, ISO, PCG, BAI, 6-MBOA, and RES (Fig. S2D). To further verify if these effects were mediated by zinc chelation, intracellular Zn^2+^ concentration was determined. Polyphenols including PA, LIQ, 4-HPAA, NCGA, ISOQ, RES, ARB, and VA significantly reduced the intracellular Zn^2+^ concentration (Fig. S2E, F), confirming their chelating capacity. In summary, based on their consistent cytoprotective efficacy across multiple concentrations and confirmed non-chelating property, four polyphenols, BAI, FIS, ISO, and MDHB, were selected as the lead non-chelating candidates for subsequent studies detailed in the main text.

**Supplementary Figure 2.** Screening for non-chelating polyphenols alleviating zinc-induced cytotoxicity in IPEC-J2 cells. (A-D) Cell viability after treatment with 5, 10, 20, and 30 μmol/L of various polyphenols (*n* = 4). (E-F) intracellular Zn^2+^ concentration following treatment with 10 μmol/L of polyphenols (*n* = 4). Results are given as mean ± SD. Bars labeled without a common letter were significantly different (*P* ＜ 0.05).

**Supplementary Figure 3.** Effects of FIS on Zn²⁺ concentrations in zinc-overloaded piglets. (A) serum Zn^2+^ concentration (*n* = 6). (B) Jejunal Zn^2+^ concentration (*n* = 6). Results are given as mean ± SD. Bars labeled without a common letter were significantly different (*P* ＜ 0.05).

**Supplementary Figure 4.** Kruskal-Wallis test bar plot of differential cecal microbiota at the genus level.

**Supplementary Figure 5.** Full unedited PVDF membranes for Figure 2D.

**Supplementary Figure 6.** Full unedited PVDF membranes for Figure 5C.

**Supplementary Figure 7.** Full unedited PVDF membranes for Figure 5F.

**Supplementary Figure 8.** Full unedited PVDF membranes for Figure 5I.

**Supplementary Figure 9.** Full unedited PVDF membranes for Figure 6J.

**Supplementary Figure 10.** Full unedited PVDF membranes for Figure 7I.

 **Supplementary Figure 11.** Full unedited PVDF membranes for Figure 7M.

**References**

[1]	Zhang L, Guan Q, Jiang J, Khan M. Tannin complexation with metal ions and its implication on human health, environment and industry: An overview. Int J Biol Macromol. 2023;253:127485. <https://doi.org/10.1016/j.ijbiomac.2023.127485>.

[2]	Al Olayan E, Aloufi A, AlAmri O, El-Habit O, Abdel Moneim A. Protocatechuic acid mitigates cadmium-induced neurotoxicity in rats: Role of oxidative stress, inflammation and apoptosis. Sci Total Environ. 2020;723:137969. <https://doi.org/10.1016/j.scitotenv.2020.137969>.

[3]	Tao Z, Hu X, Wu X, Wang Z, Shen C. Protocatechualdehyde inhibits iron overload-induced bone loss by inhibiting inflammation and oxidative stress in senile rats. International Immunopharmacology. 2024;141:13. <https://doi.org/10.1016/j.intimp.2024.113016>.

[4]	Wang Q, Xian M, Li C, Mi L, Ji J, Wu Z, et al. Protective effect of liquiritin against cisplatin-induced liver injury in mice through reduction of inflammation, oxidative stress, apoptosis and inhibition of p38 MAPK/p53 pathway based on network pharmacology and in experimental validation. Journal of Ethnopharmacology. 2026;354. <https://doi.org/10.1016/j.jep.2025.120536>.

[5]	Zhao H, Jiang Z, Chang X, Xue H, Yahefu W, Zhang X. 4-Hydroxyphenylacetic acid prevents acute APAP-induced liver injury by increasing phase II and antioxidant enzymes in mice. Frontiers in Pharmacology. 2018;9. <https://doi.org/10.3389/fphar.2018.00653>.

[6]	Cheng S, Liou C, Wu S, Lin C, Huang T, Huang W. Neochlorogenic acid ameliorates allergic airway inflammation by suppressing type 2 immunity and upregulating HO-1 expression. International Immunopharmacology. 2025;146. <https://doi.org/10.1016/j.intimp.2024.113867>.

[7]	Kowalczyk T, Piekarski J, Merecz-Sadowska A, Muskala M, Sitarek P. Investigation of the molecular mechanisms underlying the anti-inflammatory and antitumour effects of isorhapontigenin: Insights from in vitro and in vivo studies. Biomedicine & Pharmacotherapy. 2024;180. <https://doi.org/10.1016/j.biopha.2024.117479>.

[8]	Huang M, Huang W, Duan R, Huang Y, Xia Y. The repairing effect of baicalein on lead induced damage to the gut-liver axis in tadpoles. Environmental Pollution. 2025;374:1-11.

[9]	Chen M, Dai L, Fei A, Pan S, Wang H. Isoquercetin activates the ERK1/2-Nrf2 pathway and protects against cerebral ischemia-reperfusion injury in vivo and in vitro. Experimental and Therapeutic Medicine. 2017;13:1353-1359. <https://doi.org/10.3892/etm.2017.4093>.

[10]	Li S, Fan Y, Shu C, Zhou Y, Shu J. Methyl 3,4-dihydroxybenzoate alleviates oxidative damage in granulosa cells by activating Nrf2 antioxidant pathway. Journal of Ovarian Research. 2024;17. <https://doi.org/10.1186/s13048-024-01412-5>.

[11]	Yang S, Han JX, Xue M, Zhang Y, Yan A, Gao X, et al. Coixol ameliorates dopaminergic neurodegeneration by inhibiting neuroinflammation and protecting mitochondrial function. Frontiers in Pharmacology. 2025;16. <https://doi.org/10.3389/fphar.2025.1657910>.

[12]	Lei D, Sun Y, Liu J, Chi J. Bergenin inhibits palmitic acid-induced pancreatic β-cell inflammatory death via regulating NLRP3 inflammasome activation. Annals of Translational Medicine. 2022. <https://doi.org/10.21037/atm-22-3781>.

[13]	Yu T, Xie Y, Yuan J, Gao J, Xiao Z, Wu Y, et al. The nutritional intervention of resveratrol can effectively alleviate the intestinal inflammation associated with celiac disease induced by wheat gluten. Frontiers in Immunology. 2022;13. <https://doi.org/10.3389/fimmu.2022.878186>.

[14]	Domitrovic R, Rashed K, Cvijanovic O, Vladimir-Knezevic S, Skoda M, Visnic A. Myricitrin exhibits antioxidant, anti-inflammatory and antifibrotic activity in carbon tetrachloride-intoxicated mice. Chemico-Biological Interactions. 2015;230:21-29. <https://doi.org/10.1016/j.cbi.2015.01.030>.

[15]	Shu P, Wang Y, Zhang L. The effect of α-Arbutin on UVB-induced damage and its underlying mechanism. Molecules. 2024;29. <https://doi.org/10.3390/molecules29091921>.

[16]	Liu H, Yan C, Teng Y, Guo J, Liang C, Xia X. Gut microbiota and d-ribose mediate the anti-colitic effect of punicalagin in DSS-treated mice. Food & Function. 2024;15:7108-7123. <https://doi.org/10.1039/d4fo00741g>.

[17]	Hai Y, Zhang Y, Liang Y, Ma X, Qi X, Xiao J, et al. Advance on the absorption, metabolism, and efficacy exertion of quercetin and its important derivatives. Food Frontiers. 2020;1:420-434. <https://doi.org/10.1002/fft2.50>.

[18]	Zeng L, Xiang R, Fu C, Qu Z, Liu C. The regulatory effect of chlorogenic acid on gut-brain function and its mechanism: A systematic review. Biomedicine & Pharmacotherapy. 2022;149. <https://doi.org/10.1016/j.biopha.2022.112831>.

[19]	Umar M, Muzammil S, Zahoor MA, Mustafa S, Ashraf A, Hayat S, et al. Fisetin attenuates arsenic-induced hepatic damage by improving biochemical, inflammatory, apoptotic, and histological profile: In vivo and in silico approach. Evidence-Based Complementary and Alternative Medicine. 2022;2022. <https://doi.org/10.1155/2022/1005255>.

[20]	Wang Q, Sun L, Gong Z, Du Y. Veratric acid inhibits LPS-induced IL-6 and IL-8 production in human gingival fibroblasts. Inflammation. 2016;39:237-242. <https://doi.org/10.1007/s10753-015-0243-9>.

[21]	Kola A, Vigni G, Lamponi S, Valensin D. Protective contribution of rosmarinic acid in rosemary extract against copper-induced oxidative stress. Antioxidants. 2024;13. <https://doi.org/10.3390/antiox13111419>.

[22]	Lin M, Liu C, Lin Y, Hsu C. Epigallocatechin gallate modulates essential elements, Zn/Cu ratio, hazardous metal, lipid peroxidation, and antioxidant activity in the brain cortex during cerebral ischemia. Antioxidants. 2022;11. <https://doi.org/10.3390/antiox11020396>.
